# Patients’ perspectives on the COPD-GRIP intervention, a new nursing care intervention for COPD

**DOI:** 10.1186/s12875-019-0957-0

**Published:** 2019-06-10

**Authors:** Marieke Zwakman, Saskia W. M. Weldam, Sigrid C. J. M. Vervoort, Jan-Willem J. Lammers, Marieke J. Schuurmans

**Affiliations:** 1 Julius Center for Health Sciences and Primary Care, University Medical Center Utrecht, Heidelberglaan 100, 3584 CX Utrecht, the Netherlands; 2 Department of Respiratory Diseases, Division Heart & Lungs, University Medical Center Utrecht, Heidelberglaan 100, 3584 CX Utrecht, the Netherlands; 3 Cancer Center, University Medical Center Utrecht, Heidelberglaan 100, 3584 CX Utrecht, the Netherlands; 4 Julius Center for Health Sciences and Primary Care, University Medical Center Utrecht, Heidelberglaan 100, 3584 CX Utrecht, the Netherlands

**Keywords:** Chronic obstructive pulmonary disease, Nurses, Patients’ experience, Intervention, Interviews, Qualitative study

## Abstract

**Background:**

The nurse-led chronic obstructive pulmonary disease-Guidance Research on Illness Perception (COPD-GRIP) intervention was developed to incorporate illness perceptions into COPD care with the intention to improve the health-related quality of life of COPD patients. This individualized intervention focuses on identifying, discussing and evaluating illness perceptions and consists of three consultations with a practice nurse. The aim of this study is to explore patients’ experiences regarding the COPD-GRIP intervention.

**Methods:**

A qualitative interview study nested in a cluster randomized trial in primary care. One-time semi-structured individual interviews with COPD patients who were guided with the COPD-GRIP intervention were conducted. During data collection, the constant comparative approach was used. All interviews were recorded, transcribed, anonymized and uploaded to MAXQDA. To identify themes, the transcripts were independently coded by two researchers.

**Results:**

Sixteen patients were interviewed. All patients were positive and experienced an additional value of the COPD-GRIP intervention in different areas. Three main themes were identified and show that taking part in this intervention made the patients feel ‘listened to and acknowledged’, improved their awareness of the disease and its management and helped them to make lifestyle changes. Some patients suggested that the individualized care plan could be improved and to start the intervention immediately after being informed of the COPD diagnosis. All patients recommended this intervention.

**Conclusion:**

The results of this study indicate that patients acknowledge that the COPD-GRIP intervention is a useful and promising tool for providing individualized COPD care.

## Key points

- Patients with chronic obstructive pulmonary disease (COPD) receiving guidance with the COPD-GRIP intervention experienced that the practice nurse really listened to them and they felt acknowledged.
- According to the patients, their awareness of the disease and its management improved. This was explained as the awareness prevented them from avoiding their illness any longer.
- This new intervention helped the COPD patients to make lifestyle changes to decrease the burden of the COPD.

## Background

Chronic obstructive pulmonary disease (COPD) is a progressive disease characterized by dyspnoea, cough, persistent airflow limitation, and systemic features like skeletal muscle dysfunction and comorbidities [1]. COPD patients are confronted with unmet health needs, such as the need for a better understanding of their daily life limitations and the impact of COPD on their quality of life [2–4].

Illness perceptions determine to a large extent how patients respond to their illness and the above-mentioned symptoms [5]. Despite the importance of illness perceptions, these are rarely discussed within normal COPD care [6–8]. Therefore, the COPD-Guidance, Research on Illness Perception (GRIP) intervention was developed [9, 10]. This intervention translates the available evidence concerning the association of illness perceptions and the Health Related Quality of Life (HRQol) into a practical guide to provide nurse-led individualized COPD care throughout the course of the disease [9, 10].

The COPD-GRIP intervention consists of three consultations (Table 1) and is performed by practice nurses [9, 10]. These nurses are affiliated with a general practice or home care service. During the first consultation illness perceptions were assessed and discussed using the Brief Illness Perception Questionnaire (B-IPQ) [11]. Then, in the second consultation, the patients’ understanding of the relationship between their perceptions and their behaviours was discussed. Subsequently, patients were challenged to write an individualized care plan. At the last consultation, the actions that the patients had taken to change their perceptions and behaviours were evaluated [9, 10]. An English version of the booklet describing the COPD-GRIP intervention can be found on our website [12].

**Table 1:** ^a^ COPD-GRIP intervention

The COPD-GRIP intervention is applied individually for each patient and consists of three face-to-face consultations, each lasting ∼30 min. The three consultations are scheduled at ∼3-week intervals. The specific content is individualised, based on the patient’s questions, responses and the needs of the patient.	
First consultation: understanding the patient’s illness perceptions	1) Identifying and understanding patient’s illness perceptions; 2) Assessing and discussing illness perceptions using the B-IPQ as a guide for tailoring the intervention.
Second consultation: identifying the link between illness perceptions and behavior	1) Identifying the link between illness perceptions and behaviour 2) Improving patient’s understanding of the relationship between their perceptions and their behaviour, by challenging them to draw up an individual care plan (a short-term goal and a long-term goal with strategies to achieve these).
Third consultation; evaluating and discussing the individual care plan	1) Evaluation and discussion of the individual care plan. 2) Evaluating and assessing whether the individual care plan was successful and what new actions are necessary for the future. 3) If the patient did not describe a care plan, discussing actions for the future

^a^Based on and adapted with permission from Weldam, Schuurmans, Zanen, Heijmans, Sachs, Lammers (2017) [10]

In the process of evaluating such a complex intervention, it is important to explore its effects and usefulness as well as the experiences of all persons involved in the intervention [13, 14]. Such an extensive evaluation contributes to the understanding of the intervention, provides knowledge for the development of the intervention and enables the reproduction or adaption of the intervention for further implementation of new interventions [13, 14]. Therefore, we evaluated the COPD-GRIP intervention comprehensively.

The effectiveness of the COPD-GRIP intervention has been evaluated previously in a cluster randomized trial in primary care settings in the Netherlands (Netherlands Trial Registry NTR3945) and described elsewhere [10]. The primary outcome of this trial was change in health status on the Clinical COPD Questionnaire (CCQ) at 9 months. Secondary outcomes were HRQoL, daily activities, health education impact and changes in illness perceptions [10]. Additionally, the experiences of the practice nurses who applied the COPD-GRIP intervention were evaluated and are described elsewhere [9]. Notably, it is also important to evaluate patients’ experiences with this new intervention. Therefore, the present study focuses on patients’ experiences with receiving the COPD-GRIP intervention. The aim of this study was to explore the experiences regarding the COPD-GRIP intervention from patients’ perspectives and to inform health care professionals about the value and acceptability of the COPD-GRIP intervention to be used within consultations by practice nurses.

## Methods

An explorative descriptive qualitative study was performed. The qualitative study was nested within the COPD-GRIP cluster randomized trial and conducted before the results of the trial regarding the effectiveness of the COPD-GRIP intervention were known.

### Participants

Patients were purposefully sampled and were eligible to participate if they had received the COPD-GRIP intervention within the COPD-GRIP cluster randomized trial at three to 5 months before inclusion in this qualitative study. This time period was chosen to limit the risk of recall bias. To obtain a heterogeneous sample and to ensure maximum variation, diversity was sought with regard to the age, gender, level of education, disease severity and employment of patients. Patients were also sourced from different general practices for this reason.

Potential participants were identified within the database of the COPD-GRIP cluster randomized trial. The intervention arm of this cluster randomized trial included patients from 17 general practices throughout the Netherlands. To be eligible to participate in the COPD-GRIP trial, patients had to be diagnosed with mild (GOLD grade 1), moderate (GOLD grade 2), severe (GOLD grade 3) or very severe COPD (GOLD grade 4) according to the GOLD (Global initiative for chronic Obstructive Lung Disease) guidelines [1]. These guidelines include a classification of the severity of airflow limitation in COPD based on post-bronchodilator forced expiratory volume in 1 second (FEV_1_) and the forced vital capacity (FVC) [1]. The other inclusion criteria were: age of 40 years or older, lung function test performed no more than 1 year prior to enrollment, the ability to understand and read the Dutch language, and physical and mental ability to complete the questionnaires. Patients were excluded if they had a life-threatening co-morbid condition or a primary diagnosis of asthma.

Eligible patients were contacted by telephone by a researcher (MZ). If the patient was interested in participation, a patient information form was sent. After 2 weeks, the patient was asked whether he/she was still interested, and, if so, an appointment was made.

Sampling was continued until the point of saturation was reached, meaning that no new information that would improve the understanding of patients’ experiences with the COPD-GRIP intervention would be obtained [15, 16].

### Data collection

Data were collected by the use of semi-structured interviews which were held between May 2014 and December 2014. A topic list (Table 2) was used as a framework for formulating open questions. This list was based on expert knowledge and previous studies and was developed to ensure that the necessary topics were discussed during the conversation [17, 18]. The topics included experiences with the frequency and duration of the consultations, the B-IPQ questionnaire, the individualized care plan, the period from diagnosis to the initiation of the COPD-GRIP intervention and the subjects discussed during the COPD-GRIP intervention. The topic list was adapted during the process to focus on the key ideas that arose during the analysis. All interviews started with the same following question: ‘What is your experience with the three consultations that you have had within the context of the COPD-GRIP trial?’

**Table 2:** Topic list

First question	
What are your experiences with the three consultations that you have had with your practice nurse within the COPD-GRIP intervention?	
Topics	Prompt questions
The B-IPQ	What kind of difficulties did you experience while filling in the questionnaire?
The duration of the consultations	Was there enough time to discuss all the topics you wanted to discuss?
The frequency of the consultations	How was it to see your practice nurse more often in a short period of time?
The period from diagnosis to the initiation of the COPD-GRIP intervention	What would you suggest what the best moment is to participate in the COPD-GRIP intervention?
The discussed topics	In what way were the discussed topics in line with your needs?
The individual care plan	What was the result of this written individually care plan?
The added value	What did it mean for you to participate in the COPD-GRIP intervention?

*B-IPQ* Brief Illness Perception Questionnaire [11]

*COPD-GRIP* Chronic Obstructive Pulmonary Disease-Guidance, Research on Illness Perception

All interviews were conducted by a nurse scientist with experience in performing interviews (MZ). The interviews were conducted at a time and location of the patient’s choice and were audiotape-recorded. The duration of the interviews lasted approximately 45 min (range: 30–60 min). After the interview, patients’ demographics (e.g. age, gender, employment, GOLD grade and level of education) was collected with the use of a questionnaire.

### Data analysis

A thematic analysis framework was used to guide analysis [19] and was performed by two researchers (MZ and SW). Using constant comparative during the study process, allowed emerging themes to be identified in an early stage of the study and then further explored in later interviews [16].

All interviews were transcribed verbatim and anonymized. Memos were used to document initial thoughts and ideas that emerged during the coding process. The transcripts were first read out in full to get an overall picture and then were open-coded independently by two researchers (MZ and SW). During joint meetings, the two researchers compared and discussed their coding until they reached a consensus. Codes and the analytical memos were correlated to understand the meaning and to identify themes. The initial codes were then organized into categories based on thematic similarities. Subsequently, the identified codes were compared and combined to bring them under broader categories. These thematic categories and subcategories were described.

The data analysis was supported by the qualitative software MAXQDA (VERBI Software, Berlin, Germany) [20]. To enhance trustworthiness, an audit trail was performed to meet the requirements of dependability and confirmation.

## Results

Of the purposefully sampled patients (*n* = 24), 16 agreed to participate. The reasons for not taking part in the study included: no time to participate (*n* = 4), no interest (*n* = 2), no problems with COPD (*n* = 1) and not wanting to say anything negative about the nurse who performed the intervention (*n* = 1).

As presented in Table 3, the age range of the patients was 54 to 80 years, and half of the patients were female. Most of the patients had moderate COPD, four patients had severe COPD, and one patient had very severe COPD. The majority of the interviews were conducted at the patients’ homes (14 times). The other two interviews were conducted at the general practice and a neighborhood center, respectively.

**Table 3:** Patients’ demographics

		Patient n (%) or mean (range) *n* = 16
Age		
		44 years (28–58)
Gender		
	Male	8 (50%)
	Female	8 (50%)
GOLD grade^a^		
	2	11 (68,6%)
	3	4 (25%)
	4	1 (6,3%)
Employment		
	Yes	4 (25%)
	no	12 (75%)
Education^b^		
	Low	1 (6,3%)
	Medium	14 (87,5%)
	High	1 (6,3%)

^a^Gold = Global Initiative for Chronic Obstructive Pulmonary Disease, criteria classification of severity of airflow limitation [1]

^b^International standard classification of education (ISCED) sept 2011 re-edition I© UNESCO-UIS www.uis.unesco.org. 2011

- Low: junior general secondary education for adults

- Medium: vocational education, professional training diploma, senior general secondary education for adults, vocational education, middle management training diploma

- High: Bachelor

The patients’ stories revealed that they were satisfied with the COPD-GRIP intervention, especially with respect to the frequency of three consultations with the practice nurse. Many mentioned that these three consultations had an added value in managing their illness. One patient illustrated this as follows:

*‘People need guidance. They need to know they are able to slow it [COPD] down’* (female patients, 65 years of age).

Moreover, all patients stated that they would recommend the consultations to other COPD patients.

Based on patients’ experiences with the COPD-GRIP intervention, the common processes are described in three main themes: (1) being listened to and acknowledged, (2) gaining illness awareness and (3) making lifestyle changes (Table 4).

**Table 4:** Main themes, superordinate themes and subthemes that emerged from open coding, axial coding and selective coding

Main themes	Subordinate themes	Subthemes
Being listened to and acknowledged	Engagement Share Trust Duration Frequently	Cooperation Relationship Personal attention Equality Openness Encouragement Honesty Revealing true vulnerability Sincerity Open Heartedness
Gaining illness awareness	Confrontation B-IPQ	Knowledge Not running away anymore Seriousness Prognosis
Making lifestyle changes	Take action Ask for help The individual care plan The time of diagnosis disclosure and the start of the COPD-GRIP intervention	Start walking Start cycling Quite smoking Talk about the COPD Ask questions

*B-IPQ* Brief Illness Perception Questionnaire [11]

*COPD-GRIP* Chronic Obstructive Pulmonary Disease-Guidance, Research on Illness Perception

### Being listened to and acknowledged

In general, patients receiving the COPD-GRIP intervention felt that the practice nurse really listened to them and, thus, they felt acknowledged. Within the consultations of the COPD-GRIP intervention, patients experienced encouragement, trust, and an openness to share their thoughts and feelings concerning their illness. In addition, patients found that they could talk on an equal level with the practice nurse. Patients also expressed that the frequency of the consultations helped to build a confidential relationship based on trust, which allowed them to reveal their vulnerabilities. One patient clarified this with the following:

*‘Because if you met someone once a year, as it regularly is, you need time to get used to each other. That was not the case now’ (female patient, 55 years of age).*

The finding that the patients were able to reveal their vulnerabilities was supported by their reports of feeling free to express their feelings, such as embarrassment, fear, sadness, and happiness, during the consultations. Despite differences in the duration of the consultations between patients, all patients felt comfortable in asking questions when something was unclear and sharing their experiences concerning COPD (e.g. prognosis, medication, condition, fears, nutrition, quite smoking).

Most patients reported receiving personal attention during the consultations of the COPD-GRIP intervention.

*‘She [practice nurse] took time to listen to me….she really listened to me….*(female patient, 62 years of age).

‘*Yes, I liked that [COPD-GRIP consultations], because you can tell your story. You can tell how you feel and she [practice nurse] responds to it’* (male patient, 67 years of age).

The majority of the patients experienced personal engagement with the practice nurse, which they did not experience with others (e.g. friends, family, or a health care professional). This personal engagement resulted in a helpful degree of cooperation between the patient and the practice nurse and generally good adherence to the agreement they made, such as giving up smoking, increasing daily activities (e.g. walking and cycling), and expressing their feelings. One patient described this as follows: *‘If you do not succeed in giving up smoking, a friend might say “try it again tomorrow”, but with a nurse you have an agreement* (male patient, 54 years of age)*.*

Overall, the patients experienced a feeling of no longer being alone in dealing with their COPD.

### Gaining illness awareness

Patients expressed that they had become more aware of (the seriousness of) their illness. They mainly thought that this process started during the first consultation in which the B-IPQ questionnaire was completed and discussed with the practice nurse. Patients’ stories revealed that gaining illness awareness continued during the subsequent consultations with the practice nurse. These consultations provided patients both a deeper understanding and awareness of their COPD; the following example illustrates this:

*“…You did not realize, but if you get questions, for example, to what extent do you feel you understand your illness? Then there is the tendency to say yes, I understand. Whereas, while you are talking something longer about it [COPD], you can just become a little more aware of it and give it a moment’s thought”* (female patient, 55 years of age).

Patients described experiencing this illness awareness in several areas, such as the seriousness of COPD, the patient’s own prognosis and the effect of lifestyle on the progressive course of COPD. The patients stated that this awareness prevented them from avoiding their illness any longer.

Patients explained that illness awareness was a result of their increased knowledge about COPD as well as the corresponding medication and treatments, which they mentioned as being the major effect of the COPD-GRIP intervention. Although the majority of patients described an increase in their knowledge, the degree of the increase varied between patients, mainly due to the existing knowledge differences before the intervention. To illustrate, one patient expressed his increased knowledge as: *‘I became wiser and what it [COPD] is.* Later during the conversation*: I didn’t even know the word COPD’* (male patient, 80 years of age). While another patient said that the COPD-GRIP consultations only refreshed his knowledge of the subject.

According to the patients, a higher level of awareness and being aware earlier could be ensured during the COPD-GRIP intervention through confronting patients with the seriousness of COPD. For example, practice nurses could tell patients detailed stories about COPD or show pictures of COPD patients’ lungs. One patient said: *‘I feel fine, but I would like that they measure some things, at least once a year… It would be nice that I know how I am doing… but when do I feel fine? Fifty percent capacity? Sixty, seventy, eighty? That is what I would like to know’* (male patient, 59 years of age). In this regard, patients suggested that practice nurse should have comprehensive expertise in COPD to be able to answer all possible questions about COPD.

### Making lifestyle changes

Within the second consultation, patients were challenged to write an individualized care plan. This plan contains goals described by patients themselves and included often lifestyle changes.

Of the patients who wrote such an individual care plan, only some of the patients were satisfied with it. These patients believed that the completion of their individual care plan was much like a contract and, therefore, required respect and adherence.

In contrast, another patient believed that the individual care plan was too informal. Consequently, this patient suggested that the goals in the individual care plan should be formulated in a more specific manner and proposed writing the chosen goals in one’s agenda to make the individual care plan more useful and practical. Furthermore, some of the other patients mentioned that the individual care plan was simply unnecessary for them. To illustrate, one patient explained it as: *‘I think that every person need to formulate goals for himself and by himself, that is how we live… then I do it by myself and then I have to do it… it has to come from within you’* (male patient, 65 years of age). However, according to one of these patients who was not interested in completing the individual care plan, such a plan could be useful for passive patients because it might help them by drawing attention to their goals. A few patients could not remember the individual care plan and stated that it may be useful to have a copy of it to read at home now and then use as a reminder. Three patients who did receive a copy of the individual care plan during the intervention were positive about it because it reminded them to their strategies and supported them to achieve their associated goals. *‘Yes, it [a copy of the individual care plan] is rather easy. You can read over it’* (male patient, 81 years of age).

The majority of patients said that they made lifestyle changes after the consultations of the COPD-GRIP intervention. To illustrate, several patients started to improve their physical condition. Some patients began cycling, while others started to walk with their dog. A number of patients made appointments with a physical therapist for condition improvement. Some patients quit smoking after the intervention.

Additionally, a few patients mentioned that the intervention had contributed to them being able to be more open to others (e.g. friends) about their COPD, leading to social support. Other patients explained that the intervention supported them in taking more control and asking more questions to their practice nurse and doctor. In general, the consultations of the COPD-GRIP intervention revealed opportunities to the patients regarding decreasing the burden of COPD (i.e. actions that could be taken); thus, many made positive lifestyle changes and began asking for help if necessary.

According to the interviewed patients, the time between diagnosis and the start of the COPD-GRIP intervention also influenced the execution of lifestyle changes. Several patients said they would have preferred the first consultation to happen shortly after they were diagnosed with COPD. Those patients mentioned that, after COPD diagnosis, the initiation of treatment, which requires being advised about COPD, is important. In addition, one patient said: *‘I think people who just hear it [the diagnosis of COPD], because then you are working on it’* (female patient, 59 years of age). The patient who had undergone the first COPD-GRIP consultation immediately after diagnosis felt it was the correct time for that consultation. However, in contrast, a few patients stated that they would prefer to start the COPD-GRIP consultations when they experienced an increased burden of COPD because they did not want to confront the disease at an earlier stage.

## Discussion

The present study presents the experiences of COPD patients regarding the COPD-GRIP intervention with the aim of informing health care professionals about the value of this intervention from the patients’ perspectives. Generally, the patients involved in this study welcomed the consultations of the COPD-GRIP intervention and recommended them to other COPD patients. The experiences of the patients can be outlined in three main themes: (1) the COPD-GRIP intervention made the patients feel they were ‘being listened to and acknowledged’, (2) the intervention improved their “illness awareness” of the disease and its management and (3) the intervention helped them to make lifestyle changes. In addition to these positive experiences, some patients indicated that the individual care plan could be improved. Furthermore, they suggested starting the intervention directly after diagnosis, which is in line with the theory concerning the development of illness perceptions [5]. This theory describes that illness perceptions are developed directly after the patient is confronted with the diagnosis of COPD [5]. Therefore, starting the COPD-GRIP intervention by identifying and discussing illness perceptions in an earlier stage of the disease seems appropriate. Nevertheless, not every patient wants to be confronted with the disease at an early stage. Therefore, it is vital for long-term care to be tailored to the needs of each COPD patient on an individual basis.

The results of the present study show that COPD patients experience the relationship with the practice nurses as important. Moreover, patients felt personal engagement with the practice nurses based on trust and sharing their experiences. This is in line with the literature concerning the role of the nurse in primary care [4, 21, 22], especially in COPD care. This is supported by the findings of a study by Barello & Graffina [23]. The results of this study suggest that the ability of patients to engage in the care process leads to adjustments in their quality of life. In addition, a positive and trusting relationship with healthcare professionals contributes to patients’ well-being [4, 24].

As described by Smolowitz et al. [22] and Fletcher and Dahl [4], nurses play a significant role in the continuity of chronic disease management at all stages and are responsible for the quality of care. Previous research has shown that patients find it easier to talk to nurses than to doctors because they perceive nurses as having more time available [24]. Within this context, nurses perform several roles, including chronic illness case management and health coaching. These findings are consistent with the results of this qualitative study, whereas the professional engagement and professional expertise of the nurse were identified as important roles during the guidance of the COPD patient. These roles of the practice nurse require a professional attitude, meaning that nurses need to be good listeners and must have the ability to act upon their observations [22].

Some strengths and limitations of the present study should be considered when interpreting its findings. The researcher who conducted the interviews did not know the participants and by creating a nonjudgmental atmosphere, enabled them to freely express their views. Another strength of this study is the independent analysis performed by two researchers, which supports the credibility of the interpretations. Moreover, the study is strengthened by the fact that the insights that arose during the analysis process were incorporated in the interview topic list, leading to more depth. Another strength of this study is that maximum variation within the purposefully sampled participants was achieved for disease severity (grades 2–4), age (range: 54–80 years), and sex (equally distributed).

A limitation of this study is that the enrollment for this qualitative study started a half year after the start of the COPD-GRIP trial. Consequently, patients enrolled in the beginning of the aforementioned trial could not participate in this qualitative study. However, starting this qualitative study later on gave the nurses the opportunity to learn how to perform the COPD-GRIP intervention and, consequently, be more experienced in delivering the intervention.

## Conclusion

The findings of this additional study concerning the COPD-GRIP intervention represent a substantial contribution to the current understanding of this intervention. The present study provided in depth insight into patients’ experience with the COPD-GRIP intervention from their own perspective and lay bare the experienced effects of the intervention and the necessary conditions. The study revealed that an intervention based on illness perceptions is a promising tool for improving primary nursing care in COPD patients.

## Abbreviations

B-IPQ: Brief Illness perception Questionnaire
COPD: Chronic Obstructive Pulmonary Disease
COPD-GRIP: Chronic Obstructive Pulmonary Disease -Guidance, Research on Illness Perception
FEV_1_: Forced expiratory volume in one second
FVC: Forced vital capacity
GOLD: Global initiative for chronic Obstructive Lung Disease
